# Impact of a psychoeducational intervention to improve caregiver knowledge and attitudes toward palliative cancer care

**DOI:** 10.1016/j.pec.2025.109222

**Published:** 2025-06-06

**Authors:** Brenna Mossman, Lisa Molix, Damian R. Murray, Laura M. Perry, Seowoo Kim, Michael Hoerger

**Affiliations:** a Department of Psychology, Tulane University, New Orleans, LA, USA; b Cancer Prevention and Control Program, Lombardi Comprehensive Cancer Center, Georgetown University, Washington, DC, USA; c Department of Medicine, Tulane University, New Orleans, LA, USA; d Center for Health Outcomes, Implementation, and Community-Engaged Science, Tulane University School of Medicine, New Orleans, LA, USA; e Louisiana Cancer Research Center, New Orleans, LA, USA; f Department of Psychiatry, Tulane University, New Orleans, LA, USA; g Department of Palliative Medicine & Supportive Care, New Orleans, LA, USA

**Keywords:** Caregivers, Neoplasms, Oncology, Palliative care, Decision making, Health education, Attitude

## Abstract

**Objectives:**

To evaluate the efficacy of a psychoeducational intervention in improving palliative care knowledge and attitudes among caregivers, patients’ close friends and families.

**Methods:**

A total of 150 adult cancer caregivers participated in an online U.S.-based randomized clinical trial (RCT) between December 2021 and March 2022. Participants self-reported their baseline knowledge of palliative care, then viewed a psychoeducational video on palliative care (intervention condition) or nutrition (control condition). Participants then completed outcome measures of palliative care knowledge (aim 1; Palliative Care Knowledge Scale, PaCKS) and attitudes (aim 2; Palliative Care Attitudes Scale-9-Caregiver, PCAS-9C). Regression analyses examined whether group assignment significantly predicted palliative care knowledge and attitudes while controlling for self-reported baseline knowledge and key demographic and clinical characteristics.

**Results:**

The PaCKS and PCAS-9C demonstrated excellent evidence of reliability, factor structure, and validity in this caregiver sample. The RCT succeeded on the primary outcome of increasing caregivers’ palliative care knowledge (*p* < .001). Attitudes were favorable and did not differ between groups.

**Conclusions:**

This study demonstrates that a single-session, psychoeducational video can improve understanding of palliative care among close friends and families of those with cancer.

**Practice implications:**

This work suggests future pathways for helping families make informed decisions about initiating palliative care.

## 1. Introduction

A cancer diagnosis has widespread implications for both patients and their friends and families, who often adopt the critical role of caregiving [1,2]. These family caregivers provide unpaid assistance and support to those with cancer, including responsibilities such as symptom and medication management, emotional support, and healthcare system navigation, placing caregivers at risk for high levels of strain and decreased quality of life [3–9]. In line with these responsibilities, caregivers often assume important roles in medical decision-making, participating both directly and indirectly in patients’ treatment decisions [10–12]. Consequently, caregivers’ knowledge and attitudes about care may not only influence their own healthcare, but also that of the patient they care for.

This is particularly relevant for palliative care, an interdisciplinary, team-based medical specialty that supports patients and their families living with serious illness [13–16]. Research demonstrates palliative care’s ability to improve both patient and caregiver outcomes, and guidelines recommend integration into oncology care soon after diagnosis of advanced disease [13–17,5]. However, most patients never receive palliative care or receive it late, when the patient is near death [18–22]. Caregivers may contribute to this pattern of utilization, as many are unaware of palliative care or hold misconceptions about its purpose [22–25].

In non-caregiver populations, several notable studies have demonstrated the efficacy of educational interventions in improving both palliative care knowledge and attitudes. The EMPOWER studies examined these outcomes among patients with cancer in a series of two studies based on the Empowerment Theory of Palliative Care. This theory suggests that palliative care utilization relies on both cognitive and emotional processes, emphasizing the presence of a cognitive pathway (improving knowledge of palliative care) and an emotional pathway (increasing motivation to utilize palliative care) that allows patients to gain mastery over the decision to pursue palliative care [26,27]. Both the EMPOWER 1 and EMPOWER 2 studies demonstrated that educating patients about palliative care via written and video interventions, respectively, significantly improved their knowledge and attitudes towards utilization [26,27]. While another RCT has similarly improved knowledge in a non-clinical population [28], other psychoeducational interventions have not demonstrated comparable effects on palliative care knowledge in patients. Two RCTs using video-based psychoeducational interventions found no change in palliative care knowledge among women with gynecologic cancers [29] and other patients meeting palliative care referral criteria [30].

Among caregivers, there has only been one small, pilot RCT examining whether interventions are efficacious in increasing caregiver knowledge of palliative care [31]. Some non-randomized studies have demonstrated the feasibility of educating caregivers about palliative care, suggesting interventions improved caregiver knowledge [32–34]. However, internal validity was limited by the lack of randomization and a control group. Further, no known study has focused on an oncology-specific population. Thus, the evidence among patients varies, and the evidence among caregivers is primarily limited to non-RCTs.

To address these gaps in the literature, the present study implemented an online video intervention targeting palliative care knowledge and attitudes among close friends and family members of individuals with cancer. The primary aims of this study were to evaluate the effects of a psychoeducational intervention on 1) palliative care knowledge, and 2) palliative care attitudes. The intervention was grounded in prior research and was adapted from the EMPOWER 2 study that previously targeted patients [26,27]. The present study tested the hypotheses that participants who view the intervention video will gain increased knowledge and view palliative care more favorably than those in the control group.

## 2. Methods

### 2.1. Participants and recruitment

This was a single-session, anonymous online study conducted via Qualtrics between December 19, 2021, and March 26, 2022 (Tulane University Institutional Review Board #2021–1041; Clinicaltrials.gov registration NCT05162807). Individuals were eligible to participate if they provided informed consent, were 18 years of age or older, and self-reported a history of a close friend or family member diagnosed with cancer. Participants were primarily recruited through ResearchMatch, an NIH-sponsored online recruitment tool that connects interested volunteers with potential research studies [35,36]. Recruitment also occurred via postings on health- and psychology-related websites and social media pages with administrator permission. The study was also available to access through search engines, as well as email and word of mouth referrals.

### 2.2. Procedures

Following informed consent, Qualtrics’ built-in randomization feature assigned participants to the intervention or control condition. Both groups completed baseline measures of demographic and health information, health literacy, and a self-reported rating of palliative care knowledge. Participants in the intervention condition then viewed a psychoeducational video discussing palliative care, while the control group viewed an educational video on nutrition. Both the intervention and control groups then completed post-assessments of palliative care knowledge and attitudes. For data cleaning purposes, participants also confirmed whether they carefully and honestly completed the survey, whether they had completed the survey previously, and whether they or their family member/friend had ever received palliative care. After completing all assessments and submitting their responses, participants received access to both videos, as well as additional resources on palliative care, caregiving, and coping with cancer. Participants’ median study completion time was 16.5 min (*standard deviation*=22.19 min).

### 2.2.1. Intervention

The intervention arm of this study entailed viewing a psychoeducational video grounded in the Empowerment Theory of Palliative Care and was developed with the involvement of patients, their families, and clinicians [26,27]. The video introduced palliative care and discussed its purpose, structure, benefits, and efficacy using straightforward and accessible language (Flesch-Kincaid level 4.4). It was 5 min and 52 s long and suitable for all levels of education and health literacy. The video used 7 out of 10 chapters from a previously developed video that were deemed most relevant to caregivers: Welcome, Palliative Care Overview, Palliative Care Team, Typical Visit, Who Should Use Palliative Care, Caregiver Involvement, and Evidence Base [26].

### 2.2.2. Control

The control group viewed an educational video on nutrition and healthy eating during cancer survivorship [37]. Previous research on palliative care knowledge has used videos with similar content as a control [28]. The video was developed in part by the American Cancer Society, and its content was based on scientific evidence and published guidelines. Additionally, the video was similar in length (4 min and 3 s) to the intervention video, resulting in comparable time commitments for participants randomized to each group.

### 2.3. Baseline measures

#### 2.3.1. Demographic and health characteristics

Participants reported relevant demographic and health characteristics for themselves and their family member/friend at baseline. Specifically, participants reported their own age, gender, race/ethnicity, education, relationship status, financial status, health literacy, and overall perceived health. Participants also reported their family member/friend’s age, gender, race/ethnicity, whether they were living or deceased, and key health characteristics. Additionally, participants characterized their relationship with the patient by reporting how they were related, how close they were (using a numeric rating scale indicating how well they knew the patient, where 0 =not well at all and 10 =extremely well), how involved they were in the patient’s care, and whether they ever provided instrumental support (currently or in the past). Instrumental support was defined as assisting with health-related care (i.e., mobility, self-care, and/or household chores), information (i.e., gathering and communicating information), or decisions (i.e., discussing healthcare choices) [12,38,39].

#### 2.3.2. Palliative care knowledge and experience

Participants also self-reported palliative care knowledge at baseline via a single item derived from the National Institutes of Health’s Health Information National Trends Survey (HINTS) [40]. This question asked participants to indicate their level of knowledge of palliative care, with response options including “never heard of it,” “know a little bit about it, ” and “know what palliative care is and could explain it to someone else.” Prior research has used this question to assess palliative care knowledge among patients, caregivers, and the general public [41]. Participants also reported whether they or their friend/family member had ever previously received palliative care.

### 2.4. Outcome measures

#### 2.4.1. Palliative care knowledge

After viewing either the intervention or control video, participants completed the Palliative Care Knowledge Scale (PaCKS) [42]. This 13-item scale measured knowledge of palliative care through a series of questions, to which respondents answered “Yes,” “No,” or “I don’t know.” Each correct answer was scored as 1, and each incorrect or unsure answer was scored as 0. Participants’ total percentage of correct responses was then calculated to demonstrate palliative care knowledge. The PaCKS has demonstrated acceptable reliability and validity among adults [42], and it has been used in various research populations, including caregivers [32].

#### 2.4.2. Palliative care attitudes

Participants also reported their attitudes toward palliative care by completing the nine-item Palliative Care Attitudes Scale [43], adapted for use among caregivers (PCAS-9C). The PCAS-9C consisted of emotional, cognitive, and behavioral subscales with three items each. The scale asked respondents to indicate how stressful or helpful palliative care would be, as well as their willingness to attend palliative care visits.

The PCAS-9C utilized a five-point rating scale ranging from 1 to 5 throughout, and each subscale contained a unique set of response option descriptors. Subscale scores were created by summing the items within each subscale. The emotional subscale was reverse scored for consistency, so that higher scores indicated more favorable attitudes toward palliative care. Total scores were created by adding the three subscale scores. Total scores ranged from 9 to 45, with lower scores indicating more palliative care avoidance. The original measure has demonstrated acceptable reliability and validity among adults with cancer and other serious medical conditions [43], and the present study confirmed the PCAS-9C’s psychometric properties.

### 2.5. Analytic approach

#### 2.5.1. Data cleaning and screening

**Fig. 1.**
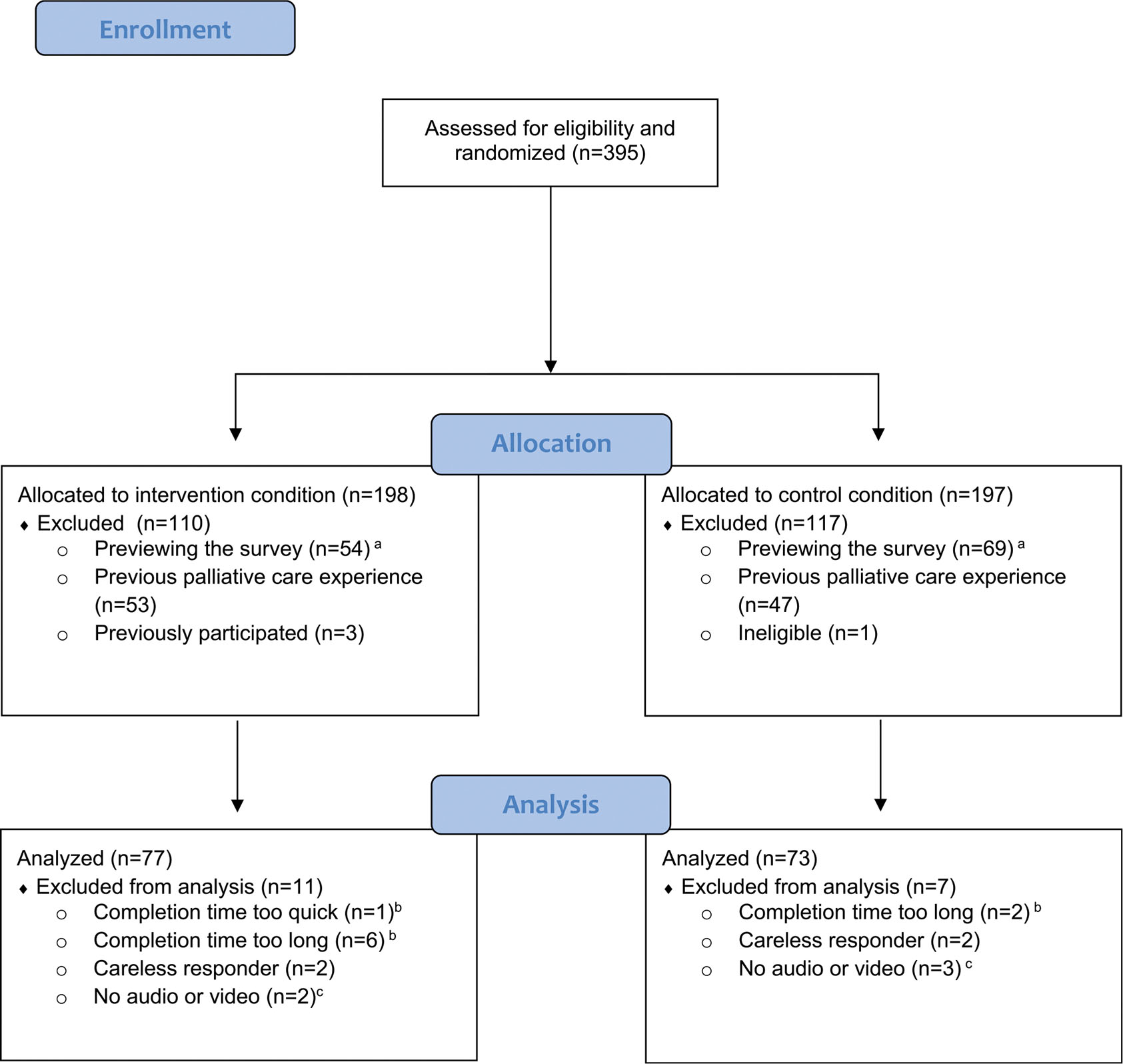
CONSORT diagram.

Prior to analyses, data were screened for response validity and missing values, assumptions for primary analyses, and psychometric performance of the outcome measures. Invalid cases were identified and removed as necessary based on time spent completing (less than the length of the condition’s video or >4 h, suggesting distractibility) or previewing the survey, and participant responses indicating their eligibility, audio/video functionality, whether they carefully and honestly completed the survey, and whether they completed the survey previously. Additionally, the analytic sample only included data from participants who indicated they or their family member/friend had never received palliative care. Fig. 1 outlines the process of sample selection, resulting in an analytic sample of 150 participants (n = 77 intervention; n = 73 control). Extreme outliers (>3 standard deviations) on the dependent variables were replaced with the closest non-outlying value prior to conducting inferential statistics [44], and we examined all necessary assumptions for the primary analysis of multiple linear regression. Additionally, psychometric analyses confirmed the outcome measures’ internal consistency and factor structure in the present sample.

#### 2.5.2. Statistical analyses

Descriptive analyses first characterized the sample via summary statistics and zero-order Pearson bivariate correlation coefficients for demographic and health characteristics, palliative care knowledge, and scores on the outcome measures. T-tests and chi-square tests determined any baseline differences between the intervention and control groups.

For hypothesis testing, multiple linear regression assessed the effect of intervention vs. control group assignment on PaCKS and PCAS-9C scores. Regression analyses utilized two separate regression models: one with PaCKS total percent correct as the dependent variable and one with PCAS-9C total scores as the dependent variable. Each model included group assignment as the independent variable and the following predictors as covariates: self-reported baseline knowledge of palliative care assessed via a separate single item (present vs. absent), age, gender (male vs. other), education (Bachelor’s degree present vs. absent), instrumental support (providing vs. not providing instrumental support), patient cancer type (dummy coded for the two most common diagnoses), time since diagnosis, cancer stage (metastases present vs. absent), and patient vital status (living vs. deceased). Within the models, the covariate of palliative care knowledge at baseline was dummy coded as present if the participant indicated they knew what palliative care was and could explain it to someone else. Covariates were selected based on clinical relevance and past research indicating their importance [26, 27,45]. For this primary analysis of multiple linear regression, the study was powered to detect an effect size of *f*^*2*^= .15 as statistically significant (α=.05) at 90 % for the entire model and 99 % for individual coefficients within the model (two-tailed).

Following primary analyses, we conducted a set of sensitivity analyses to further examine and clarify any effects of the intervention. First, any additional demographic and health variables that were significantly correlated with PaCKS or PCAS-9C total scores or any variables that differed across groups at baseline were added as covariates to the primary regression models described above. Additionally, we added level of closeness to the patient as a covariate and examined the interaction between baseline knowledge of palliative care and the intervention in the primary regression models.

## 3. Results

A total of 150 participants comprised the analytic sample, with 77 participants in the intervention and 73 participants in the control condition. Table 1 presents descriptive characteristics for the intervention group, control group, and total sample. Participants (i.e., caregivers) had a mean age of 49.66 (*standard deviation [SD]*=16.19) and were primarily female (*n* = 125, 83.3 %) and educated with a Bachelor’s degree or higher (*n* = 114, 76.0 %). Participants predominantly reported being White and non-Hispanic or Latino/a (*n* = 121, 80.7 %), with the next most frequently reported race/ethnicities being Black or African American (*n* = 12, 8.0 %) and Hispanic or Latino/a (*n* = 7, 4.7 %). Participants were most commonly patients’ adult children (*n* = 60, 40.0 %) or spouses/romantic partners (*n* = 34, 22.7 %). Most participants provided instrumental support (*n* = 117, 78.0 %) and were at least “a little involved” in the patient’s medical care (*n* = 128, 85.3 %). At baseline, participants self-reported that they never heard of palliative care (*n* = 27, 18.0 %), knew a little bit about it (*n* = 60, 40.0 %,), or knew what palliative care was and could explain it someone else (*n* = 63, 42.0 %).

Caregivers also reported patients’ demographic and health information. Patients were more often female (*n* = 88, 58.7 %), and their mean current age or age at death was reportedly 63.37 years old (*SD*=14.94). Patients were most frequently reported as White and non-Latino/a (*n* = 120, 80.5 %), followed by Black or African American (*n* = 12, 8.0 %) and Hispanic or Latino/a (*n* = 7, 4.7 %). Patients were most commonly diagnosed with breast (*n* = 41, 27.3 %) or gastrointestinal (*n* = 27, 18.0 %) cancers, and they were diagnosed an average of 10.30 (*SD*=11.84) years ago. A total of 32.0 % (*n* = 48) of patients had metastatic cancer, and 66.0 % (*n* = 99) of patients were currently living. Among both caregiver and patient characteristics, the intervention and control groups only differed with respect to patient receipt of chemotherapy (intervention: *n* = 42, 54.5 %; control: *n* = 53, 72.6 %; *p* = .022). Intervention and control groups were not significantly different on any other demographic, health, or treatment-related variables. A follow-up sensitivity analysis adjusted for this difference by including chemotherapy as a covariate in regression to examine any effects of this difference on knowledge and attitudes [27].

### 3.1. Knowledge and attitudes: descriptive statistics and bivariate associations

Table 2 characterizes the PaCKS and PCAS-9C outcome scores. Scores on the PaCKS ranged from 0 to 100 percent correct (*mean [M]*= 84.00, *SD*=24.75). The intervention group answered a mean of 91.8 % of items correctly (*SD*=15.10). The control group answered a mean of 75.8 % of items correctly (*SD*=29.89). Scores were significantly different across groups (*p* < .001), with a medium-to-large effect of *d*= −.68. Approximately 92.2 % of the intervention group scored above the control group’s mean PaCKS score. Scores on the PCAS-9C ranged from 21 to 44 (*M*=34.77, *SD*=4.36), where higher scores indicate more favorable attitudes towards palliative care. Mean scores for each group were similar, with intervention and control group means of 34.73 (*SD*=4.12) and 34.82 (*SD*=4.64), respectively (*d*=.022, *p* = .895). Psychometric analyses confirmed the internal consistency and factor structure of the PaCKS (α=.914; RMSEA=.037; TLI=.989; SRMR=.083; CFI=.991) and the three-factor structure of the PCAS-9C (α=.765; RMSEA=.066, TLI=.960; SRMR=.062; CFI=.973).

Table 3 presents associations between intervention group assignment, model covariates, and palliative care knowledge (PaCKS) and attitudes (PCAS-9C). Palliative care knowledge was significantly correlated with the intervention (*r* = .335, *p* < .001), caregiver self-reported baseline palliative care knowledge (*r* = .394, *p* < .001), and caregiver age (*r* = .214, *p* = .008). Palliative care attitudes were significantly correlated with caregiver gender (*r* = −.230, *p* = .005), with women holding more favorable palliative care attitudes. Furthermore, palliative care knowledge and attitudes were significantly correlated with one another (*r* = .211, *p* = .010).

### 3.2. Hypothesis testing

Table 4 presents the results of the multiple linear regression analyses predicting palliative care knowledge on the PaCKS total scores (model 1) and attitudes on the PCAS-9C total scores (model 2). As hypothesized for the first aim, the intervention increased knowledge of palliative care (β=.309, *p* < .001), even when controlling for baseline self-reported knowledge and demographic and health covariates. Among these covariates, baseline self-reported knowledge of palliative care (β=.332, *p* < .001) and caregiver age (β=.174, *p* = .023) significantly predicted outcome knowledge on the PaCKS. Sensitivity analyses demonstrated that the effect was particularly strong for those with limited self-reported palliative care knowledge at baseline, as evidenced by the interaction between the intervention and baseline knowledge item (β=−.192, *p* = .004). The intervention did not affect palliative care attitudes (β=−.002, *p* = .983). Among covariates, only caregiver gender (β=−.237, *p* = .005) was significant, with males having less favorable attitudes towards palliative care. This pattern of findings held even when controlling for additional variables within the sensitivity analyses (see Table 4).

## 4. Discussion and conclusion

### 4.1. Discussion

This is the first known RCT to demonstrate that a video-based educational intervention can increase knowledge of palliative oncology care among patients’ close friends and family members. The present study was an online, single-session RCT evaluating the impact of a psychoeducational video intervention on palliative care knowledge and attitudes among caregivers, defined within this study as the close friends and family members of people with a history of cancer. Results found that the intervention was associated with better performance on a validated assessment of palliative care knowledge administered shortly after the video, and this finding remained even when controlling for pre-existing self-reported knowledge and other relevant demographic and health covariates. Given the intervention’s strong influence on palliative care knowledge and its brief and accessible format, these findings have important implications for education within clinical settings and promoting communication among clinicians, patients, and family caregivers.

This research was innovative in that it is among the first known RCTs aiming to improve knowledge of palliative care among patients’ close friends and family members. One other pilot RCT has examined the impact of a broader, self- and family-management intervention on knowledge of care options among caregivers, finding that palliative care literacy significantly improved among the intervention group, but the sample size was small (N = 35) and constrained to breast cancer caregivers [31]. To the best of our knowledge, all other previous studies examining caregiver palliative care education have been uncontrolled, focusing only on pretest-posttest changes in a single group [32,33,46] or level of knowledge based on unrandomized community exposure [34]. Further, these studies have either utilized non-cancer samples [32], not differentiated between cancer caregivers and other serious illness caregivers [33], or focused exclusively on bereaved family members of those with cancer [34] or those caring only for patients currently undergoing chemotherapy [46]. Moreover, these studies employed more cumbersome interventions consisting of various educational materials (e.g., verbal presentations paired with videos or written materials), ranging from approximately 30-minute sessions [32,33,46] to three-year public educational campaigns [34]. Thus, this study is unique in terms of its randomization and control group, its cancer-specific focus and inclusion of close friends and family members who may have been currently providing care, and its sole use of a brief, single-material video intervention, which significantly reduces barriers to implementation. This intervention format is easily adaptable to clinical workflows, allowing for cost effective, standardized use that can be further evaluated in clinical settings in future studies.

More broadly, this study also helps to clarify the overall impact of video-based interventions on palliative care knowledge across various populations. Prior RCTs in non-caregiver populations have found both significant and null effects of videos on palliative care knowledge [26]. While one RCT among laypersons demonstrated a relatively small but significant effect of a video intervention on knowledge [28], two other video-based RCTs among patients found no significant effect on knowledge [29,30]. The present research reveals strong evidence of palliative care knowledge improvement with a psychoeducational video, further supporting the potential utility of this accessible intervention format for educational purposes. Furthermore, based on the evidence from the present study, our video intervention appears to be suitable for a range of populations that vary considerably in terms of health literacy and education. It may be especially important for clinical application in lower literacy populations, as the intervention effect was particularly strong among those with limited baseline palliative care knowledge. This is notable for implementation, as this video would ideally be provided shortly after diagnosis of advanced disease, a time when patients and caregivers may know little about palliative care.

This was also the first known RCT to examine palliative care attitude improvement among patients’ close friends and family members. However, contrary to hypotheses, the intervention did not significantly improve palliative care attitudes. This null finding may be due to a number of reasons. First, the sample had overall positive attitudes towards palliative care, a potential reflection of self-selection bias in this sample, and the manipulation may not have been strong enough to improve the sample’s already-favorable attitudes. Second, while the intervention video was accessible to all education and health literacy levels, this study’s highly educated sample may have benefitted from a more nuanced version of the intervention that discussed palliative care in greater depth. Third, the original intervention video contained more chapters than were included in the present study. Some of these chapters had important information that may have influenced attitudes, but they weren’t well-suited to the current study and were thus removed. Lastly, researchers originally designed the video to improve patients’ attitudes. It is possible that caregivers may have separate fears or reservations about palliative care than patients, and the intervention – while still informative about palliative care’s purpose, structure, and benefits – may not have adequately addressed these concerns. Future research may wish to further emphasize caregiver-specific benefits of palliative care and assess different facets of caregivers’ attitudes towards palliative care utilization.

#### 4.1.1. Strengths and limitations

This study is strengthened by its novelty, brief intervention format, and use of a control group. However, it is limited by the relatively homogenous sample in terms of race, ethnicity, gender, culture, and education, restricting generalizability. Future studies should recruit more representative samples with respect to sociodemographic groups that may be sensitive to differential palliative care attitudes and utilization [47–49]. Additionally, the study design did not include pre-test assessments of the outcomes, which may have allowed for the analysis of differential changes among the intervention and control groups. Utilizing a single baseline knowledge item reduced participant burden and the risk of the intervention influencing participants’ baseline responses (access previous/future content in the survey was not restricted), but it limited our analyses and ability to estimate pre-post effect sizes. Furthermore, study outcomes were assessed shortly after viewing the psychoeducational video. Additional research is needed to determine whether participants retain palliative care knowledge over time. Lastly, participants differed in their level of involvement in patients’ healthcare. While caregivers may provide support outside of direct involvement in healthcare [7], 22 % of the sample did not provide instrumental support, and we did not assess current levels of caregiving involvement or intensity. The intervention may ultimately be more impactful among those who are currently more actively involved in patients’ medical care and decision-making, especially those actively caring for patients who are eligible for palliative care. Subsequent studies should aim to examine knowledge and attitude improvement in current caregivers, particularly those caring for a patient recently diagnosed with advanced cancer, as this would be the ideal time to implement the intervention.

#### 4.1.2. Future directions

Moving forward, future research should aim to further elucidate the relationship between caregivers’ knowledge and attitudes towards palliative care. This research may benefit from using more caregiver-specific psychoeducational materials that are tailored to their perspectives, concerns, and behaviors. Survey-based research exploring caregiver-specific barriers to palliative care utilization would be helpful. Further, future studies should examine whether interventions result in sustained improvements in knowledge or attitudes over time in a more representative sample of current caregivers, and whether increased understanding of palliative care ultimately translates to increased utilization among both patients and caregivers.

## 5. Conclusion

This is among the first known RCTs to demonstrate the efficacy of an intervention to improve caregiver knowledge of palliative care. This work has implications for the use of video interventions in clinical settings, particularly among those with lower health literacy. Future work should further explore the relationship between knowledge, attitudes, and utilization, ultimately aiming to increase palliative care utilization and improve patient and caregiver quality of life.

### Practice implications

This study utilized a short, one-time video to effectively educate caregivers about palliative care, which is a scalable design conducive to use in clinical settings. Specifically, this brief video format may be a helpful and effective conversational aid that allows patients, caregivers, and clinicians to discuss palliative care and other end-of-life care preferences. Prior work has shown that caregivers and patients rarely have conversations with their clinicians about end-of-life care preferences, including palliative care [50–53]. If these conversations do occur, the discussion is often postponed until the patient is close to death [54,55]. However, caregivers report a desire for increased communication about this topic, including routine education about palliative care before the time of referral [56]. Therefore, this video intervention may be able to prompt discussion among patients, caregivers, and clinicians, enabling timelier referral to palliative care and, ultimately, medical care that more closely aligns with patients’ wishes.

## Figures and Tables

**Table 1:** Caregiver and patient baseline characteristics.

Characteristic	M (SD) or *n* (%)			*p* ^a^
	Intervention (*n* = 77)	Control (*n* = 73)	Total (N = 150)	

**Caregiver characteristics**				
Age, years	49.78 (17.15)	49.53 (15.22)	49.66 (16.19)	.927
Gender, male	14 (18.2 %)	10 (13.7 %)	24 (16.0 %)	.454
Race/Ethnicity, White and non-Latino/a	65 (84.4 %)	56 (76.7 %)	121 (80.7 %)	.232
Education level, Bachelor’s or higher	58 (75.3 %)	56 (76.7 %)	114 (76.0 %)	.842
Marital status, married	42 (54.5 %)	43 (58.9 %)	85 (56.7 %)	.590
Financial strain, present	17 (22.1 %)	21 (28.8 %)	38 (25.3 %)	.346
Location				.975
Southern U.S.	25 (32.5 %)	23 (31.5 %)	48 (32.0 %)	
Western U.S.	21 (27.3 %)	22 (30.1 %)	43 (28.7 %)	
Midwestern U.S.	21 (27.3 %)	17 (23.3 %)	38 (25.3 %)	
Northeastern U.S.	8 (10.4 %)	9 (12.3 %)	17 (11.3 %)	
International	2 (2.6 %)	2 (2.7 %)	4 (2.7 %)	
Relationship to patient				.905
Adult child/child-in-law	31 (40.3 %)	29 (39.7 %)	60 (40.0 %)	
Spouse/partner	17 (22.1 %)	17 (23.3 %)	34 (22.7 %)	
Sibling/Sibling-in-law	8 (10.4 %)	8 (11.0 %)	16 (10.7 %)	
Friend	9 (11.7 %)	6 (8.2 %)	15 (10.0 %)	
Grandchild	6 (7.8 %)	7 (9.6 %)	13 (8.7 %)	
Niece/Nephew	4 (5.2 %)	2 (2.7 %)	6 (4.0 %)	
Other	2 (2.6 %)	4 (5.5 %)	6 (4.0 %)	
Closeness to patient, 0–10 rating^b^	9.06 (1.69)	9.11 (1.57)	9.08 (1.63)	.826
Provided instrumental support^c^	60 (77.9 %)	57 (78.1 %)	117 (78.0 %)	.981
Duration of instrumental support provision, years	3.33 (6.42)	2.83 (5.02)	3.09 (5.76)	.641
Involvement in healthcare, 1–5 rating^d^	3.16 (1.41)	3.18 (1.33)	3.17 (1.36)	.921
Health literacy, 0–20	18.44 (2.66)	18.74 (1.74)	18.59 (2.26)	.420
Baseline knowledge of palliative care, present^e^	36 (46.8 %)	27 (37.0 %)	63 (42.0 %)	.226
Perceived health, 1–5 rating^f^	3.88 (.86)	3.85 (.92)	3.87 (.87)	.817
**Patient characteristics**				
Age, years^g^	63.94 (16.14)	62.76 (13.63)	63.37 (14.94)	.634
Gender, male	32 (41.6 %)	30 (41.1 %)	62 (41.3 %)	.954
Race/Ethnicity, White and non-Latino/a	62 (80.5 %)	58 (79.5 %)	120 (80.5 %)	.870
Cancer diagnosis				
Breast	22 (28.6 %)	19 (26.0 %)	41 (27.3 %)	.727
Gastrointestinal	14 (18.2 %)	13 (17.8 %)	27 (18.0 %)	.953
Genitourinary	17 (22.1 %)	8 (11.0 %)	25 (16.7 %)	.068
Lung, head, and neck	13 (16.9 %)	10 (13.7 %)	23 (15.3 %)	.588
Hematologic	9 (11.7 %)	11 (15.1 %)	20 (13.3 %)	.543
Gynecologic	2 (2.6 %)	6 (8.2 %)	8 (5.3 %)	.159
Brain	2 (2.6 %)	6 (8.2 %)	8 (5.3 %)	.159
Skin	3 (3.9 %)	5 (6.8 %)	8 (5.3 %)	.486
Other	4 (5.2 %)	7 (9.6 %)	11 (7.3 %)	.302
Time since diagnosis (years)	9.7 (11.58)	10.93 (12.16)	10.30 (11.84)	.527
Treatment types				
Chemotherapy	42 (54.5 %)	53 (72.6 %)	95 (63.3 %)	.022
Surgery	47 (61.0 %)	45 (61.6 %)	92 (61.3 %)	.939
Radiation	41 (53.2 %)	35 (47.9 %)	76 (50.7 %)	.516
Metastases present	22 (28.6 %)	26 (35.6 %)	48 (32.0 %)	.355
Comorbidities present	43 (55.8 %)	40 (54.8 %)	83 (55.3 %)	.897
Vital status, living	55 (71.4 %)	44 (60.3 %)	99 (66.0 %)	.149

a *p*-values derived from chi-square tests (categorical variables) and t-tests (continuous variables)

b Participants indicated how well they knew their friend/family member on a 0-10 numeric rating scale, where 0 = Not well at all and 10 = Extremely well

c Instrumental support was defined as providing assistance via health-related care, information, or decision-making

d Participants rated how involved they were in the patient’s healthcare on a 1-5 scale, with response options “Not involved at all,” “A little involved,” “Somewhat involved,” “Very involved,” and “Extremely involved”

e Knowledge of palliative care was considered present at baseline if participants indicated that they “know what it is and could explain it to someone else”

f Participants rated their perceived overall health on a scale from 1 (Poor) to 5 (Excellent)

g Patient’s current age or age at death, if deceased

**Table 2:** Palliative care knowledge and attitude outcome scores.

Outcome	Score Range^a^	Mean (SD)				
		Intervention	Control	Total	Cohen’s d	p-value^b^

Knowledge, PaCKS total percent correct	0–100	91.81 (15.10)	75.76 (29.89)	84.00 (24.75)	−.68	< .001
Attitudes, PCAS-9C total score	21–44	34.73 (4.12)	34.82 (4.64)	34.77 (4.36)	.022	.895

*Note.* PaCKS = Palliative Care Knowledge Scale. PCAS-9C = Palliative Care Attitudes Scale - Caregiver version.

a Possible scores range from 0 to 100 for the PaCKS and 9–45 for the PCAS-9C.

b Unadjusted comparison of post-test means calculated via *t*-test.

**Table 3:** Correlation between group assignment, covariates, and palliative care knowledge and attitudes.

Variable	1	2	3	4	5	6	7	8	9	10	11	12

1. Palliative care knowledge^a^	.914^a^											
2. Palliative care attitudes^b^	.211**	.765^a^										
3. Intervention	.335***	−.013	-									
4. Caregiver baseline palliative care knowledge	.394***	.131	.099	-								
5. Caregiver age	.214**	.115	.008	.050	-							
6. Caregiver gender, male	−.043	−.230**	.061	−.187*	−.010	-						
7. Caregiver education	.058	−.083	−.016	.194*	−.114	−.095	-					
8. Caregiver instrumental support	.142	.081	−.002	.061	.238**	.012	−.110	-				
9. Breast cancer	−.127	−.093	.029	−.007	−.025	−.104	.134	−.144	-			
10. Gastrointestinal cancer	.077	.000	.005	.023	−.014	−.062	−.021	.039	−.209	-		
11. Time since diagnosis	.038	.098	−.052	−.093	.185*	.039	−.061	−.031	.019	.004	-	
12. Metastases present	.111	.061	−.075	.053	.023	−.027	−.183*	.088	−.196*	.237**	.004	-
13. Patient vital status	.076	−.053	.118	.183*	−.109	.006	.124	.060	.156	−.140	−.503**	−.202*

a Cronbach’s alpha for outcome measures are presented for the control group. Alphas for the intervention group are less meaningful indicators of internal consistency due to response to the intervention.

*** Significant at p < .001

** Significant at p ≤ .01

* Significant at p < .05

**Table 4:** Multiple linear regression: impact of intervention on palliative care knowledge and attitudes.

	*Model 1 (Knowledge* ^a^ *)*		*Model 2 (Attitudes* ^b^ *)*	
Predictors	β (95 % CI)	p-value^c^	β (95 % CI)	p-value

Intervention	.309 (.168,.451)	< .001	−.002 (−.164.160)	.983
Covariates				
Caregiver baseline knowledge of palliative care^d^	.326 (.196,.456)	< .001	.105 (−.065,.276)	.223
Caregiver age	.174 (.025,.323)	.023	.066 (−.103,.234)	.442
Caregiver gender, male	−.007 (−.189,.176)	.943	−.237 (−.401, −.072)	.005
Caregiver education^e^	.057 (−.093,.208)	.454	−.092 (−.261,.077)	.283
Caregiver instrumental support	.059 (−.100,.217)	.466	.039 (−.129,.206)	.649
Cancer type				
Breast	−.117 (−.289,.055)	.181	−.108 (−.278,.062)	.212
Gastrointestinal	.031 (−.119,.181)	.681	−.045 (−.212,.123)	.600
Time since diagnosis	.103 (−.081,.286)	.270	.110 (−.079,.299)	.251
Metastases present	.105 (−.040,.249)	.154	.020 (−.153,.192)	.822
Patient vital status, living	.084 (−.110,.278)	.394	.016 (−.182,.213)	.875

*Note.* Model 1 R^2^= .320; Model 2 R^2^= .108. Sensitivity analyses added the following covariates: patient receipt of chemotherapy, caregiver race and ethnicity, caregiver financial strain, closeness to the patient, caregiver health literacy, and patient gender. Caregiver age was no longer significant in Model 1, but the effects of the intervention and baseline knowledge of palliative care remained significant at *p* < .001. The pattern of findings in Model 2 did not change.

a Based on Palliative Care Knowledge Scale (PaCKS) total scores. Higher scores indicate more knowledge of palliative care.

b Based on Palliative Care Attitudes Scale - Caregiver (PCAS-9C) total scores. Higher PCAS-9C scores indicate more favorable attitudes towards palliative care.

c *p*-values and confidence intervals based on robust standard errors adjusting for heteroskedasticity.

d Based on a single self-reported item assessing general knowledge of palliative care.

e Presence of a Bachelor’s degree or higher.

## References

[1] de Moor JS, Dowling EC, Ekwueme DU, Guy GP Jr, Rodriguez J, Virgo KS, Employment implications of informal cancer caregiving. J Cancer Surviv 2017;11:48–57. 10.1007/s11764-016-0560-5. 27423439 PMC5239760

[2] Ochoa CY, Buchanan Lunsford N, Lee Smith J. Impact of informal cancer caregiving across the cancer experience: a systematic literature review of quality of life. Palliat Support Care 2020;18:220–40. 10.1017/S1478951519000622. 31588882 PMC8678891

[3] Kehoe LA, Xu H, Duberstein P, Loh KP, Culakova E, Canin B, Quality of life of caregivers of older patients with advanced cancer. J Am Geriatr Soc 2019;67:969–77. 10.1111/jgs.15862. 30924548 PMC7818364

[4] Trevino KM, Prigerson HG, Maciejewski PK. Advanced cancer caregiving as a risk for major depressive episodes and generalized anxiety disorder. Psychooncology 2018;27:243–9. 10.1002/pon.4441. 28426918 PMC5746474

[5] Hoerger M, Cullen BD. Early integrated palliative care and reduced emotional distress in cancer caregivers: reaching the “hidden patients”. Oncologist 2017;22:1419–20. 10.1634/theoncologist.2017-0432. 28982800 PMC5728035

[6] Ullgren H, Tsitsi T, Papastavrou E, Charalambous A. How family caregivers of cancer patients manage symptoms at home: a systematic review. Int J Nurs Stud 2018;85:68–79. 10.1016/j.ijnurstu.2018.05.004. 29857223

[7] Tolbert E, Bowie J, Snyder C, Bantug E, Smith K. A qualitative exploration of the experiences, needs, and roles of caregivers during and after cancer treatment: “that’s what I say. I’m a relative survivor”. J Cancer Surviv 2018;12:134–44. 10.1007/s11764-017-0652-x. 29101710

[8] Duimering A, Turner J, Chu K, Huang F, Severin D, Ghosh S, Informal caregiver quality of life in a palliative oncology population. Support Care Cancer 2020;28:1695–702. 10.1007/s00520-019-04970-3. 31292753

[9] Kent EE, Mollica MA, Buckenmaier S, Wilder Smith A. The characteristics of informal cancer caregivers in the United States. Semin Oncol Nurs 2019;35:328–32. 10.1016/j.soncn.2019.06.002. 31229342

[10] Lamore K, Montalescot L, Untas A. Treatment decision-making in chronic diseases: what are the family members’ roles, needs and attitudes? A systematic review. Patient Educ Couns 2017;100:2172–81. 10.1016/j.pec.2017.08.003. 28838630

[11] Laidsaar-Powell R, Butow P, Charles C, Gafni A, Entwistle V, Epstein R, The TRIO framework: conceptual insights into family caregiver involvement and influence throughout cancer treatment decision-making. Patient Educ Couns 2017;100:2035–46. 28552193 10.1016/j.pec.2017.05.014

[12] Dionne-Odom JN, Ejem D, Wells R, Barnato AE, Taylor RA, Rocque GB, How family caregivers of persons with advanced cancer assist with upstream healthcare decision-making: a qualitative study. PLoS One 2019;14:e0212967. 10.1371/journal.pone.0212967. 30865681 PMC6415885

[13] Ferrell BR, Temel JS, Temin S, Smith TJ. Integration of palliative care into standard oncology care: ASCO clinical practice guideline update summary. J Oncol Pr 2017;13:119–21. 10.1200/JOP.2016.017897. 28972832

[14] El-Jawahri A, Greer JA, Pirl WF, Park ER, Jackson VA, Back AL, Effects of early integrated palliative care on caregivers of patients with lung and gastrointestinal cancer: a randomized clinical trial. Oncologist 2017;22:1528–34. 10.1634/theoncologist.2017-0227. 28894017 PMC5728034

[15] Hoerger M, Wayser GR, Schwing G, Suzuki A, Perry LM. Impact of interdisciplinary outpatient specialty palliative care on survival and quality of life in adults with advanced cancer: a Meta-Analysis of randomized controlled trials. Ann Behav Med 2019;53:674–85. 10.1093/abm/kay077. 30265282 PMC6546936

[16] Alam S, Hannon B, Zimmermann C. Palliative care for family caregivers. J Clin Oncol 2020;38:926–36. 10.1200/JCO.19.00018. 32023152

[17] Rogers JL, Perry LM, Hoerger M. Summarizing the evidence base for palliative oncology care: a critical evaluation of the Meta-analyses. Clin Med Insights Oncol 2020;14:1179554920915722. 10.1177/1179554920915722. 32341671 PMC7171985

[18] Scibetta C, Kerr K, McGuire J, Rabow MW. The costs of waiting: implications of the timing of palliative care consultation among a cohort of decedents at a comprehensive cancer center. J Palliat Med 2016;19:69–75. 10.1089/jpm.2015.0119. 26618636

[19] den Herder-van der Eerden M, van Wijngaarden J, Payne S, Preston N, Linge-Dahl L, Radbruch L, Integrated palliative care is about professional networking rather than standardisation of care: a qualitative study with healthcare professionals in 19 integrated palliative care initiatives in five european countries. Palliat Med 2018;32:1091–102. 10.1177/0269216318758194. 29436279 PMC5967037

[20] Han X, Shi KS, Zhao J, Nogueira L, Parikh RB, Kamal AH, Medicaid expansion associated with increase in palliative care for people with Advanced-Stage cancers. Health Aff (Millwood) 2023;42:956–65. 10.1377/hlthaff.2023.00035. 37406229

[21] Pini S, Hackett J, Taylor S, Bekker HL, Kite S, Bennett MI, Patient and professional experiences of palliative care referral discussions from cancer services: a qualitative interview study. Eur J Cancer Care (Engl) 2021;30:e13340. 10.1111/ecc.13340. 33051957

[22] Bandieri E, Borelli E, Gilioli F, Bigi S, Mucciarini C, Ferrari U, Stigma of palliative care among patients with advanced cancer and their caregivers on early palliative care. Cancers (Basel) 2023;15. 10.3390/cancers15143656. 37509317 PMC10377431

[23] Collins A, McLachlan SA, Philip J. Initial perceptions of palliative care: an exploratory qualitative study of patients with advanced cancer and their family caregivers. Palliat Med 2017;31:825–32. 10.1177/0269216317696420. 28367679

[24] Dionne-Odom JN, Ornstein KA, Kent EE. What do family caregivers know about palliative care? Results from a national survey. Palliat Support Care 2019;17:643–9. 10.1017/S1478951519000154. 30957733 PMC6783327

[25] Zimmermann C, Swami N, Krzyzanowska M, Leighl N, Rydall A, Rodin G, Perceptions of palliative care among patients with advanced cancer and their caregivers. E27 CMAJ 2016;188:E217. 10.1503/cmaj.151171. PMC4938707 27091801

[26] Perry LM, Sartor O, Malhotra S, Alonzi S, Kim S, Voss HM, Increasing readiness for early integrated palliative oncology care: development and initial evaluation of the EMPOWER 2 intervention. J Pain Symptom Manag 2021. 10.1016/j.jpainsymman.2021.03.027. PMC8526633 33864847

[27] Hoerger M, Perry LM, Gramling R, Epstein RM, Duberstein PR. Does educating patients about the early palliative care study increase preferences for outpatient palliative cancer care? Findings from project EMPOWER. Health Psychol 2017;36:538–48. 10.1037/hea0000489. 28277698 PMC5444973

[28] Kozlov E, Reid MC, Carpenter BD. Improving patient knowledge of palliative care: a randomized controlled intervention study. Patient Educ Couns 2017;100:1007–11. 10.1016/j.pec.2016.12.022. 28034612 PMC5879772

[29] Graul A, Haggerty A, Stickley C, Kumar P, Morales K, Bogner H, Effect of patient education on palliative care knowledge and acceptability of outpatient palliative care services among gynecologic oncology patients: a randomized controlled trial. Gynecol Oncol 2020;156:482–7. 10.1016/j.ygyno.2019.11.023. 31831167

[30] Kamal AH, Wolf S, Nicolla JM, Friedman F, Xuan M, Bennett AV, Usability of PCforMe in patients with advanced cancer referred to outpatient palliative care: results of a randomized, Active-Controlled pilot trial. J Pain Symptom Manag 2019;58:382–9. 10.1016/j.jpainsymman.2019.05.007. PMC10230646 31163259

[31] Schulman-Green D, Linsky S, Blatt L, Jeuland J, Kapo J, Jeon S. Improving breast cancer family caregivers’ palliative care literacy: a pilot randomized trial. J Fam Nurs 2023;29:99–114. 10.1177/10748407221099541. 35670155

[32] Noh H, Lee LH, Won C. Educational intervention to improve palliative care knowledge among informal caregivers of cognitively impaired older adults. Palliat Support Care 2020:1–9. 10.1017/S1478951520001200. 33234188

[33] Cruz-Oliver DM, Malmstrom TK, Fernandez N, Parikh M, Garcia J, Sanchez-Reilly S. Education intervention “caregivers like me” for latino family caregivers improved attitudes toward professional assistance at End-of-life care. Am J Hosp Palliat Care 2016;33:527–36. 10.1177/1049909115584315. 26019262

[34] Akiyama M, Hirai K, Takebayashi T, Morita T, Miyashita M, Takeuchi A, The effects of community-wide dissemination of information on perceptions of palliative care, knowledge about opioids, and sense of security among cancer patients, their families, and the general public. Support Care Cancer 2016;24:347–56. 10.1007/s00520-015-2788-4. 26076961

[35] Harris PA, Scott KW, Lebo L, Hassan N, Lightner C, Pulley J. ResearchMatch: a national registry to recruit volunteers for clinical research. Acad Med 2012;87:66–73. 10.1097/ACM.0b013e31823ab7d2. 22104055 PMC3688834

[36] Tan MH, Thomas M, MacEachern MP. Using registries to recruit subjects for clinical trials. Conte Clin Trials 2015;41:31–8. 10.1016/j.cct.2014.12.012. PMC4380621 25545027

[37] ACS. Eating well during and after treatment. Society AC, ed. YouTube; 2014.

[38] Monin JK, Levy B, Doyle M, Schulz R, Kershaw T. The impact of both spousal caregivers’ and care recipients’ health on relationship satisfaction in the caregiver health effects study. J Health Psychol 2019;24:1744–55. 10.1177/1359105317699682. 28810439 PMC5786494

[39] Polenick CA, Leggett AN, Webster NJ, Han BH, Zarit SH, Piette JD. Multiple chronic conditions in spousal caregivers of older adults with functional disability: associations with caregiving difficulties and gains. J Gerontol B Psychol Sci Soc Sci 2020;75:160–72. 10.1093/geronb/gbx118. 29029293 PMC6909432

[40] Nelson D, Kreps G, Hesse B, Croyle R, Willis G, Arora N, The health information national trends survey (HINTS): development, design, and dissemination. J Health Commun 2004;9:443–60. 15513791 10.1080/10810730490504233

[41] Adjei Boakye E, Mohammed KA, Osazuwa-Peters N, Lee MJ, Slomer L, Emuze D, Palliative care knowledge, information sources, and beliefs: results of a national survey of adults in the USA. Palliat Support Care 2020;18:285–92. 10.1017/S1478951519000786. 31571557

[42] Kozlov E, Carpenter BD, Rodebaugh TL. Development and validation of the palliative care knowledge scale (PaCKS). Palliat Support Care 2017;15:524–34. 10.1017/S1478951516000997. 28025952

[43] Perry LM, Hoerger M, Malhotra S, Gerhart JI, Mohile S, Duberstein PR. Development and validation of the palliative care attitudes scale (PCAS-9): a measure of patient attitudes toward palliative care. e8 J Pain Symptom Manag 2020;59:293–301. 10.1016/j.jpainsymman.2019.09.008. PMC12449757 31539604

[44] Sadiq S handbook of data quality: research and practice. Heidelberg, Germany. Springer; 2013.

[45] Bazargan M, Cobb S, Assari S, Kibe LW. Awareness of palliative care, hospice care, and advance directives in a racially and ethnically diverse sample of california adults. Am J Hosp Palliat Care 2021;38:601–9. 10.1177/1049909121991522. 33535787

[46] Ibrahim AM, Elnaghy SF, Abo Elmatty GM, Mohamed Ghida NI, Mohamed MA. Effectiveness of a palliative care education program for caregivers of cancer patients receiving chemotherapy in port said city: a pre-post quasi-experimental study. Palliat Support Care 2024;22:546–62. 10.1017/S1478951523002067. 38287515

[47] Mossman B, Perry LM, Walsh LE, Gerhart J, Malhotra S, Horswell R, Anxiety, depression, and end-of-life care utilization in adults with metastatic cancer. Psychooncology 2021;30:1876–83. 10.1002/pon.5754. 34157174

[48] Gardner DS, Doherty M, Bates G, Koplow A, Johnson S. Racial and ethnic disparities in palliative care: a systematic scoping review. Fam Soc J Contemp Soc Serv 2018;99:301–16.

[49] Saeed F, Hoerger M, Norton SA, Guancial E, Epstein RM, Duberstein PR. Preference for palliative care in cancer patients: are men and women alike?. e1 J Pain Symptom Manag 2018;56:1–6. 10.1016/j.jpainsymman.2018.03.014. PMC6015521 29581034

[50] Brighton LJ, Bristowe K. Communication in palliative care: talking about the end of life, before the end of life. Post Med J 2016;92:466–70. 10.1136/postgradmedj-2015-133368. 27153866

[51] Knutzen KE, Sacks OA, Brody-Bizar OC, Murray GF, Jain RH, Holdcroft LA, Actual and missed opportunities for End-of-Life care discussions with oncology patients: a qualitative study. JAMA Netw Open 2021;4:e2113193. 10.1001/jamanetworkopen.2021.13193. 34110395 PMC8193430

[52] Trevizan FB, Paiva CE, Juliao M, de Oliveira Valentino TC, Miwa MU, Mingardi M, Comprehension and Decision-Making capacity questionnaire about palliative care and advance care planning: a delphi study. J Palliat Care 2023;38:41–51. 10.1177/08258597221128676. 36168276

[53] Manz CR, Zhang Y, Chen K, Long Q, Small DS, Evans CN, Long-term effect of machine Learning-Triggered behavioral nudges on serious illness conversations and End-of-Life outcomes among patients with cancer: a randomized clinical trial. JAMA Oncol 2023;9:414–8. 10.1001/jamaoncol.2022.6303. 36633868 PMC9857721

[54] El-Jawahri A, Nelson AM, Gray TF, Lee SJ, LeBlanc TW. Palliative and End-of-Life care for patients with hematologic malignancies. J Clin Oncol 2020;38:944–53. 10.1200/JCO.18.02386. 32023164 PMC8462532

[55] Kuusisto A, Santavirta J, Saranto K, Korhonen P, Haavisto E. Advance care planning for patients with cancer in palliative care: a scoping review from a professional perspective. J Clin Nurs 2020;29:2069–82. 10.1111/jocn.15216. 32045048

[56] Collins A, McLachlan SA, Philip J. How should we talk about palliative care, death and dying? A qualitative study exploring perspectives from caregivers of people with advanced cancer. Palliat Med 2018;32:861–9. 10.1177/0269216317746584. 29235421

